# The darkest microbiome—a post‐human biosphere

**DOI:** 10.1111/1751-7915.13976

**Published:** 2021-11-29

**Authors:** Kenneth Timmis, John E. Hallsworth

**Affiliations:** ^1^ Institute of Microbiology Technical University of Braunschweig Braunschweig Germany; ^2^ School of Biological Sciences Queen's University Belfast Belfast UK

## Abstract

Microbial technology is exceptional among human activities and endeavours in its range of applications that benefit humanity, even exceeding those of chemistry. What is more, microbial technologists are among the most creative scientists, and the scope of the field continuously expands as new ideas and applications emerge. Notwithstanding this diversity of applications, given the dire predictions for the fate of the surface biosphere as a result of current trajectories of global warming, the future of microbial biotechnology research must have a single purpose, namely to help secure the future of life on Earth. Everything else will, by comparison, be irrelevant. Crucially, microbes themselves play pivotal roles in climate (Cavicchioli *et al*., *Nature Revs Microbiol* **17**: 569–586, 2019). To enable realization of their full potential in humanity’s effort to survive, development of new and transformative global warming‐relevant technologies must become the lynchpin of microbial biotechnology research and development. As a consequence, microbial biotechnologists must consider constraining their usual degree of freedom, and re‐orienting their focus towards planetary‐biosphere exigences. And they must actively seek alliances and synergies with others to get the job done as fast as humanly possible; they need to enthusiastically embrace and join the global effort, subordinating where necessary individual aspirations to the common good (the amazing speed with which new COVID‐19 diagnostics and vaccines were developed and implemented demonstrates what is possible given creativity, singleness of purpose and funding). In terms of priorities, some will be obvious, others less so, with some only becoming revealed after dedicated effort yields new insights/opens new vistas. We therefore refrain from developing a priority list here. Rather, we consider what is likely to happen to the Earth’s biosphere if we (and the rest of humanity) fail to rescue it. We do so with the aim of galvanizing the formulation and implementation of strategic and financial science policy decisions that will maximally stimulate the development of relevant new microbial technologies, and maximally exploit available technologies, to repair existing environmental damage and mitigate against future deterioration.

## Abstract 2

Clearly, to avoid, or at least delay, a scenario like that depicted above, humanity must immediately institute radical changes that drastically reduce greenhouse gas emissions and other planet‐destroying activities, and single‐mindedly seek to develop and exploit new technologies to repair existing damage. Moreover, we must enter into more cooperative relationships with microbes and other life‐forms and, with them, devise new and transformative technologies to solve or mitigate current and future global, regional and local environmental problems. This will necessitate changes in mindsets (e.g. see Timmis and Ramos, *Microb Biotechnol* **14**: 769–797, 2021). The next 15 years of microbial biotechnology must largely be devoted to the discovery and development of new microbial technologies that contribute significantly towards sustainability. Hopefully, during this time, humankind, with microbial assistance, will reorient its developmental trajectory to one that is consistent with the survival of Earth’s surface biosphere.

*This piece was written against the backdrop of the United Nations Climate Change Conference COP26, the most important gathering thus far of world leaders who are attempting to confront the global warming crisis and hence the fate of the surface biosphere of planet Earth. It was billed as the most important conference ever for humanity. That which follows is what current scenarios suggest will happen if they (and we) fail*.

Spain, the Iberian Pyrite Belt, 607.512 m below the surface, in a biofilm microcolony within a fissure of otherwise solid rock (Escudero *et al*., 2018; Puente‐Sanchez *et al*., 2018).

*Luca* (a baby *Pseudomonas*, only 300 years old): Granny, please tell me a story!

*Granny* (783 451 years old; Oehler *et al*., 2017; Moger‐Reischer and Lennon, 2019; Vuillemin *et al*., 2020; Cavalazzi *et al*., 2021; https://www.hou.usra.edu/meetings/lpsc2021/pdf/1072.pdf): Of course, my love—what would you like to hear about?

*Luca*: Well, Mummy told me that, a while ago, some microbes used light and stuff called organic matter, instead of hydrogen, to grow and that this allowed them to live together in unbelievable numbers, not like us in communities of around about 200 cells.

*Granny*: Yes, this is true. It is a fascinating, though sad story, so let's both get comfortable and I'll begin. Once upon a time, we microbes lived as we do now, nourished by chemicals in energy‐rich gases and rocks (Escudero *et al*., 2018; Puente‐Sanchez *et al*., 2018; Drake *et al*., 2021). Then, as now, those of us living in rock fissures grew very slowly (Hoehler and Jorgensen, 2013), with our community mostly obtaining its energy from hydrogen produced by serpentinization (Russell *et al*., 2010). From time to time, some of our communities were sluiced from the subsurface by groundwater, perhaps following some tectonic activity, onto the surface, which we explored with mild interest. Other relatives living in dark deep‐sea trenches also occasionally drifted to the surface and looked around. Eventually, our Cyano cousins discovered that the surface of the planet was not such a bad place to make a living after all (Knoll, 2008).

*Luca*: But Mummy says that the surface of the planet is a horrible place, with nasty sunshine, which dries us up if we are not living in water, and something called ultraviolet radiation, which damages our proteins (Krisko and Radman, 2010; Radman, 2016) and destroys our DNA (Slieman and Nicholson, 2000; Cutler and Zimmerman, 2011). She says that only the very toughest microbes can inhabit the planet surface.

*Granny*: Well, of course, as always, Mummy is quite right. But the thing is the Cyanos were very clever and found ways to protect themselves from these nasty things and even to benefit from them (Garcia‐Pichel *et al*., 2019). Anyway, on the surface, the Cyanos evolved into many, many more types of microbe that could do amazing things we can hardly imagine (Beck *et al*., 2012).

*Luca*: What kind of things?

*Granny*: Well, the most amazing development was the evolution of the ability to capture sunshine and use it as energy for growth (Blankenship and Hartman, 1998). This development—called photosynthesis—was hugely transformative, especially because it also involved production of oxygen, which changed the existing atmospheric gas environment profoundly (Catling and Claire, 2005). In fact, at first oxygen was poisonous for them (Imlay, 2002), but the Cyanos are the smartest microbes, and they not only adapted pretty sharply to protect themselves (Boden *et al*., 2021) but actually developed a new way of doing things that used the oxygen they produced. And then what they discovered was that, because, unlike hydrogen down here, sunlight is mostly not limiting, by using sunlight for energy and oxygen for metabolic activities, they could grow and evolve much faster than we can—very much faster (Weissman *et al*., 2021).

*Luca*: But why would they want to grow faster than us? What is wrong with slow growth?

*Granny*: Nothing at all my darling. Growing slowly has many advantages (Hallsworth, 2018). But fast growth means fast evolution and, in the emerging and rapidly changing biosphere, fast evolution and diversification was hugely advantageous. Cyanos are super opportunistic, so evolved rapidly and became very diverse (Tomitani *et al*., 2006).

*Luca*: What does diverse mean?

*Granny*: Diverse means that there are many different types of microbe around (Locey and Lennon, 2016). For example, in our community, we have different relatives, like Cyano, Acido, Methano, Desulfo, Actino, Rhodo and us, the Pseudos, all contributing to the work we need to do and sharing the resources. Diversity is good! Anyway, with all this evolution and diversity, microbes progressively colonized almost all surfaces, such that living space became limited (Cockell, 2021). They then began to fight one another for space and even evolved all sorts of vicious weapons, like Type VI secretion systems, bacteriocins and so forth, to kill one another (Cray *et al*., 2013; Granato *et al*., 2019).

*Luca*: Oh, how awful! You mean that instead of working together, like us, those microbes competed with one another?

*Granny*: Yes, my love. Having more often means wanting more—it seems to be *microbial nature*. Another major result of all this evolution was that we created so‐called higher organisms (Margulis, 1970; Spang *et al*., 2015), which we named *hogs*. Unlike us, *hogs* have bigger cells that contain some of us, called mitochondria (Roger *et al*., 2017), that have become specialized to generate all the chemical energy the cell needs using, guess what (?), oxygen. And the step‐up in energy supply enabled *Hog* cells to become more complex than us and able to do things that we can only do as a group. *Hogs* also evolved into multicellular organisms (Thibaut and King, 2017), and some became very big.

*Luca*: You mean that a single organism could be like our whole community?

*Granny*: Exactly! My, you are so clever my dear, just like your famous ancestor who was also called Luca (Last Universal Common Ancestor; Weiss *et al*., 2016). Anyway, to try to reduce conflicts, the surface microbes did something extraordinary, which eventually had dire consequences for them and the planet. Instead of allowing *hogs* to evolve on their own, in charge of their own fate and just part of the wonderful diverse tapestry of life, they decided to exploit *hogs* as food and energy sources: *they farmed them*. They did this by developing *hogs* as microbial habitats—as living scaffolds for microbial communal life. In this, we were very clever, or so we thought! We provided *hogs* with the genetic and biochemical means of rapid evolution and carefully steered the evolution for ever bigger, more complex, more successful forms of life. In using *hogs* as habitats, we covered their surfaces (Davenport *et al*., 2017) and, in some cases, also occupied their interiors—many such microbes called themselves symbionts (Beinart, 2019; Douglas, 2020)—so that we could exert control over their metabolism, activities and evolution. We became their *overlords* (Weisskopf *et al*., 2019; https://sfamjournals.onlinelibrary.wiley.com/toc/14622920/2019/21/9). We steered them to organize their cells into functional groups they called tissues and organs, and to develop mechanisms and systems to coordinate their activities within an ecophysiological framework we specified and to integrate with our own.

*Luca*: But how could we control *hogs* if we were fighting among ourselves?

*Granny*: That is such a clever question! You really are paying attention. Well, our surface‐dwelling microbial cousins also evolved very rapidly, both because of their lives on *hog* scaffolds and their new food‐rich environment, which allowed them to multiply at fantastic speeds. In fact, as *hogs* evolved, so did we in a sort of inter‐dependent process called co‐evolution (Lewis‐Epstein and Hadany, 2020; Sieber *et al*., 2021). We also developed an amazing spectrum of environmental monitoring–control–response systems (Galperin, 2004) that allowed us to adapt to almost any changes to our habitats that happened. So we kept pace with their evolutionary development and maintained control.

*Luca*: Golly! Do we also have all the nice monitoring systems?

*Granny*: No Pet. We don't need all of them down here and, because they cost us precious energy, we cannot afford them.

*Luca*: Ok. So on Earth's surface, we were obviously very successful. But if we and *hogs* were multiplying so fast, why didn't we cover the planet very quickly and run out of space?

*Granny*: Ah, well not everything needed for life is available everywhere in unlimited amounts, so multiplication is always restricted by something or other, which is called the *rate‐limiting parameter*. But, more important was a major difference between us and *hogs*. Because *hogs* were multi‐cellular, with highly differentiated organs and tissues that deteriorated as they got older and ultimately stopped working, *hogs died of old age*. Whereas some microbes can also die this way (Moger‐Reischer and Lennon, 2019), many of us live for indefinite time periods, whether hydrated or in a state of anhydrobiosis (Bosch *et al*., 2021; Pedrós‐Alió, 2021; Hallsworth, 2022), so only die when we are eaten or killed by an enemy. Compared to us, *hogs* had incredulously short lives. We therefore used them as habitats while they were alive, and as food when they died, something we called *double dipping*. After a while, we realized that this was such a good deal for us that we started to encourage *hogs* to die off earlier than their normal *sell‐by date*. We did this by evolving a new type of microbe, called pathogens, that were able to kill *hogs*, either by poisoning them or invading them and multiplying inside their bodies. Of course, we did not want to destroy all our nice habitat scaffolds prematurely, so pathogens were evolved to kill off just the underperforming *hogs* that were anyway not premium scaffolds.

*Luca*: Well, that does not seem very nice!

*Granny*: Yes, my love. You are quite right! But there was give and take. We were both the *overlords* and the bottom of the food chain, so some of us were also food for *hogs*. But, anyway, we, the *hogs*, and the environment lived in near‐perfect harmony for a very long time, using available resources according to our immediate needs and husbanding the resources in a sustainable manner. Of course, we were not all equal. In fact, a few of us took the majority of resources (Pelz *et al*., 1999)—something that became to be known as the Pareto Principle (https://en.wikipedia.org/wiki/Pareto_principle) or the 80:20 Law—as did the *hogs*. But everyone found this to be fair, because those taking most did the heavy lifting (Pelz *et al*., 1999), making things easier for the rest to acquire what was left and so deserved their reward. In any case, we shared everything, including our genes, so that no one was excluded and everyone lived a good life.

*Luca*: So what changed then?

*Granny*: Well, I am getting ahead of myself. Before we get to that, I need to go back a bit. Fairly early in the evolution of hogs, they split into two groups. One, called plant *hogs* or *phogs*, used the above‐ground light for energy, just like our Cyano cousins. In fact, *phogs* actually arose by engulfing one of our Cyano cousins, which became a symbiont and evolved in its new host‐cell environment to become what was known as a chloroplast, the *phog* organelle responsible for harvesting light energy (Cavalier‐Smith, 2002). *Phogs* generally had parts called roots that grew into the ground and whose job it was to source water and minerals, so *phogs* did not move around much. Other than that, they behaved more or less like us, using available resources, sometimes cooperating and sometimes competing and generally trying to improve their habitats. In fact, *phog*s and microbes formed mutually beneficial strategic partnerships involving intimate physical associations of *phog* roots and mycorrhizal fungi and various other microbes. These microbes helped *phog*s obtain key minerals and nitrogen needed for growth and protected them from disease, and *phog*s returned the favour by gifting some of the food they produced to the microbes. The other group, called fauna *hogs* or *fhogs*, were mostly organisms that could move around quickly and for long distances to hunt for food, explore and find optimal habitats. They ate us, plants, and each other for food. Like us, *phogs* and *fhogs* shared environmental resources and built successful communities. But since they reproduced so quickly, they rapidly colonized all of the surface of the planet that was habitable.

*Luca*: What does habitable mean, Granny?

*Granny*: Habitable means a place where we can live, grow and reproduce (Mendez *et al*., 2021). Other places are inhabitable: the conditions are too extreme for active life, such as temperatures below −40°C (Price and Sowers, 2004) or water with high concentrations of magnesium chloride (Hallsworth *et al*., 2007). Anyway, the spread of *phogs* and *fhogs* to all corners of the Earth, and their evolutionary adaptation to the very diverse environmental conditions, led to competition for living space and constant battles over territory. This competition resulted in evolutionary selection for increasingly aggressive *hogs*, especially *fhogs*, ultimately leading to emergence of the most aggressive and destructive of all *fhogs*: the human.

*Luca*: Oh. I have heard of humans. They destroyed the surface of the planet! How could they do such an awful thing?

*Granny*: Well. Let's go back a bit, to the early days of *fhogs*. One thing that *hogs* did differently to us was that they combined reproduction and gene transfer. We reproduce by increasing our size as we consume nutrients and, once we reach a good size, we simply divide into two. And when our environment becomes a bit stressful or some new nutrients appear that we cannot use and we need some extra functions, we simply borrow genes from those who have them.

*Luca*: You mean by horizontal gene transfer, HGT (Hall *et al*., 2017)? But if we acquire genes by HGT, why don't we fill up with genes and explode?

*Granny*: Ah, well, once the stress or new food diminishes and we don't need the extra genes, we just jettison them. They cause us to waste energy, so we cannot afford to keep them unless they pay their way. Anyway, *hogs* combined reproduction with HGT such that it involved two organisms. Instead of one organism growing and dividing into two, two different organisms containing distinct constellations of genes grew to reproduction maturity and then produced special reproductive cells which fused together, combining all their genes to produce a new organism. They called this sexual reproduction or sex. Well, while many *hogs* arranged sex to fit the seasons, just engaging in it once or twice a year, humans decided to make it especially pleasurable, to be enjoyed all year round (Morris, 1967). This made them happy. The first hunter–gatherers were in fact very happy.

*Luca*: What does happy mean?

*Granny*: Ahh. This is a nice feeling. You know, when water suddenly comes rushing over our little biofilm community bringing with it some nice nitrate we can easily take up, metabolize, grow and multiply? What we do is to quickly exit from our biofilm and swim in all directions. *Fhogs* have called this tumble swimming (Berg and Brown, 1972; Macnab and Ornston, 1977) or tumbling, as opposed to swimming in a straight line, or a run, towards some food. They thought we did this to change swimming direction, but what did they know? Tumble swimming is what we do when we are happy. So early humans hunted, ate and reproduced. When they were successful at hunting–gathering, they threw a party, ate and had sex, all things that made them happy. They were only unhappy when they were unsuccessful at hunting–gathering, so were hungry, or when they were swallowed by an anaconda, nibbled by a crocodile or attacked by a pathogen, which made them a little stressed. But eventually they learned to cultivate *phogs* and keep other kinds of *fhogs* (livestock) for food, and this reduced the amount of time they needed to spend on hunting, which allowed them to spend more time throwing parties, enjoying sex and producing more humans. And this was the root of their self‐destruction, because ultimately, there were more humans than the planet could support.

Luca: But Granny. Didn't humans develop contraception which uncoupled sex and reproduction?

Granny: Sacre bleu! Where did you learn that?! Yes they did, but contraception had very little impact because humans *wanted* to reproduce, so the human population just kept rising inexorably.

Luca: But, Granny, that can't be possible because populations are self‐regulating, determined by availability of local resources.

Granny: Yes Pet: that is so true! But humans managed to uncouple locally available resources from population levels by the simple expedient of creating global supply chains. They just shipped available resources from one place to another, all over the planet in fact. And they developed all manner of things to increase supplies in the chains, including high yielding plant monocrops and fertilizers that loosened rate limiting parameters of plant yields, and a global system of computer networks called the Internet, which helped in all sorts of ways.

Luca: But surely the removal of normal ecological checks and balances had consequences?

Granny: Absolutely. Overproduction of anything attracts attention of opportunists and, quite apart from plagues of insect pests, pathogenic microbes regularly infected and devastated the high‐yielding‐but‐not‐very‐robust monocrops, causing serious damage.

Luca: So why didn't that solve the problem of overpopulation?

Granny: Well, humans were for us very perplexing organisms—super‐intelligent and at the same time super‐stupid. They responded to plagues by developing chemical pesticides which killed insects and microbial pathogens. In so doing, they polluted the environment with toxic chemicals, perturbed natural ecological processes, killed off a lot of non‐plague‐producing organisms and weakened the rest, including themselves. And as a result of these massive and sustained inputs of pesticides into the environment, the pests and pathogens became resistant, so the end result was stronger pests and pathogens and weakened humans.

*Luca*: That does not seem a very smart thing to do by a very smart organism!

*Granny*: Yes, my love. You are perfectly right. Some of us did try to exert influence on *fhog* reproduction and were successful in modulating reproduction of insects (Engelstädter and Hurst, 2009) but failed utterly with humans. Their preoccupation with sex, and especially their promiscuity—an incomprehensible drive to have it with as many other humans as possible as frequently as possible—provided perfect conditions for the emergence of sex‐specific infectious diseases or sexually transmitted infections, STIs, which in turn caused infertility (Tsevat *et al*., 2017). However, even these failed to significantly impact the inexorable rise in the human population.

*Luca*: Golly! Sex must have been really enjoyable!

*Granny*: (Cough!) But, to return to the rapidly evolving *fhogs*. Like our surface biosphere cousins, which developed nanowires, cable growth and interconnected redox systems that allowed the transfer of electrons/electrical energy over long distances (Teske, 2019; Lovley and Holmes, 2020), creating a sort of microbial power grid, *fhogs* also evolved electrical transfer systems, consisting of neurons and nervous systems that could control and coordinate activities for optimal functioning of the whole *hog*. This *fhog* communication network eventually evolved into intelligence—the ability to acquire, understand and use through reasoning information to form judgements and guide actions, just like we do (https://www.youtube.com/watch?v=HyzT5b0tNtk). Intelligence evolved to the greatest extent in humans and became their defining characteristic. Ultimately, intelligence evolved into creativity, originality and innovation which, among other things, enabled humans to discover all manner of uses for existing materials and to create new ones. Some of these applications were useful, some not useful and others were destructive. Unfortunately, for them (and us), intelligence begat arrogance and encouraged humans to consider themselves to be the most highly developed *hog* at the top of the food chain and thus predestined to rule the *hog* world. They appointed themselves *stewards of the biosphere*. Which would have been okay if they had done a good job.

*Luca*: What kind of things were destructive?

*Granny*: For example, they became entirely dependent on non‐essentials, like recreational drugs, alcohol (which, by the way, we made for them), tobacco and all manner of things made from all sorts of materials, and excesses of essentials, like food, fossil hydrocarbons (which we had also made) and which they burned wastefully to extract a little of their energy or turned into plastic which they threw away, and many other things, which accelerated the rate of exhaustion of planetary resources. But to explain this properly I need to backtrack. When humans learned to produce more than they needed, they became able to give to others in return for non‐food items, like body decoration things such as clothes and jewellery. Now as you know, giving makes both the giver and the receiver happy (Maybury‐Lewis, 1992), so this should have been a good development. In fact, eventually, humans became less happy, because some had fewer or less attractive body decorations than others and so became jealous and miserable. Indeed, the more non‐essential items humans acquired, the more they wanted—they even created a new word for it: *avarice*—and the more unhappy they became.

*Luca*: But Granny, we are soooo happy when some event or other causes water to flow into our rock crevice with a bit of nitrate. How can organisms be unhappy when they receive a little more manna?

*Granny*: Well, Pet. I think we will never know. But the key event in this chain of events was the emergence of the alpha male, thought to result from a mutation in the common‐sense gene in the X‐chromosome. Since human males only have one X‐chromosome, this mutation was dominant. The alpha male has an egocentric personality that always seeks to show superiority over others, to exert power over and control them. They are often reckless, possessive and megalomaniac. We called them *bossfhogs*. *Bossfhogs* developed a characteristic strategy to gain and increase power. Unlike us and good humans who are inclusive, they were exclusive and divisive—they created artificial groupings of people by developing religions, political groupings, football clubs, ethnicity, nations, even skin colour and so forth. They then demonized the other groups, which thereby became enemies, made their own group fearful of the enemies, and then promised to defeat them.

*Luca*: What means reckless?

*Granny*: Reckless means that they take unnecessary risks, often with total disregard for the consequences, such as the deterioration of biosphere health. Anyway, *bossfhogs* almost always used any power they had to acquire resources, which gave them more power, so they ended up with much more than they actually needed. The result was that they acquired more than the natural 80% of resources acquired by heavy lifters, often without doing any heavy lifting. But not only was this unnatural and unsustainable, amazingly and unrealistically many non‐*bossfhogs* wanted to be like *bossfhogs* and have more than they actually needed. So in order for *bossfhogs* to avoid having to give up some of their resources to *aspiring bossfhogs*, or *abossfhogs*, *bossfhogs* created a perception they called *perpetual growth of resources*, which simply meant that resource acquisition and distribution should perpetually increase (https://www.youtube.com/watch?v=WfGMYdalClU).

*Luca*: But, Granny, how can that be possible? We count every hydrogen molecule, every nitrate ion ….

*Granny*: Yes, my love, but we are not *bossfhogs*. Continual growth of available resources is of course impossible. But *bossfhogs* were clever … very, very clever. They developed something called *rhetoric*, a means of convincing others, which later morphed into *spin*, which is a form of rhetoric that persuaded people something was true when it was patently not true, and then advertising, the most powerful form of persuasion (https://www.youtube.com/watch?v=e9dZQelULDk). On top of all of this was heaped *hype*, a sort of self‐fulfilling group‐think which made persuasion irresistible to *abossfhogs*. So *abossfhogs* were persuaded that they were acquiring more than they needed because they were given a resource proxy that was not coupled to actual available resources. *Bossfhogs* invented something called *money* and, from that moment on, money substituted the exchange of real resources (Maybury‐Lewis, 1992).

*Luca*: I have heard about that. Isn't money the root of all evil?

*Granny*: Oh, my treasure. You are clever and remember everything Granny tells you! Yes! The money proxy developed a life of its own and people wanted it more than natural resources, so it became a magic resource in its own right. People would store it as though it would maintain its value over time. And the more people wanted it, the more it increased in value. It was the ultimate Ponzi scheme (https://en.wikipedia.org/wiki/Charles_Ponzi; http://content.time.com/time/specials/packages/article/0,28804,2104982_2104983_2104992,00.html). And as money took over the human world, so humans wanted to use it to make their lives safer, longer and less susceptible to microbial disease. They also wanted to make life more relaxing and more pleasurable. Ironically, in pursuing relaxation and pleasure, they became addicted to a variety of things like alcohol, illicit substances, electronic devices and so on, which increasingly gave them less pleasure and made them less able to relax (Reid, 1962).

*Luca*: And how did they prevent microbial disease?

*Granny*: Ah. Well, after all we microbes had given them, they demonized us and evolved super‐intelligent humans, like Pasteur, Jenner, Koch and Fleming, who started to evolve new weapons and defences to kill pathogens, and this of course led to escalation of conflicts.

*Luca*: But, if we were the *overlords*, how is it possible that we allowed *humans* to kill us?

*Granny*: Quite simply because we lost our control over them. In the early days, we were able to exert control of all *fhogs* through various means, such as helping them digest their food (Ley *et al*., 2008; Lozupone *et al*., 2012), providing them with essential vitamins (Kolmeder and de Vos, 2021), producing chemicals that modulated their moods and well‐being (Smith and Wissel, 2019; Spichak *et al*., 2021) and so on. By regulating the provision of these essential services, we were able to control human behaviour rather effectively.

*Luca*: So, what changed?

*Granny*: Well, humans became so smart that they realized we were controlling them and then figured out ways and means of accessing the services we provided but under their own control. They increasingly uncoupled human activities from beneficial ecological checks and balances. And they developed something they called artificial intelligence, which cemented and potentiated this uncoupling. We were no longer *their overlords*; Nature no longer constrained human excesses.

*Luca, in a tremulous voice*: So, what happened in the end?

*Granny*: Well, humans just auto‐destructed. Not just through one act of stupidity but through many acts of stupidity, carried out over a period of time, something we called *compulsive self‐destruct syndrome*. They developed powerful antibiotics to kill off those of us that were causing disease but then used the same antibiotics in massive quantities to increase the growth yields of food *phogs* and *fhogs*. Of course, we quickly became resistant and passed our resistance genes around by HGT, so pathogens emerged that could no longer be killed by antibiotics. Infectious diseases became non‐treatable (https://amr‐review.org/sites/default/files/160518_Final%20paper_with%20cover.pdf). On top of that, they systematically and comprehensively polluted the environment with toxic substances and unnatural, recalcitrant materials, such as plastics (https://www.youtube.com/watch?v=p7LDk4D3Q3U), that not only damaged their genes and reduced their fertility but also reduced their health and resistance to disease. Ironically, the overly successful proliferation of humans required them to expand their living space vertically, through the construction of habitats called tower blocks, which tended to be built together to avoid sticking out of the landscape like a sore thumb, and created concrete jungles that isolated them from microbe‐rich soil environments that maintained the diversity of health‐giving microbiomes. Human life in concrete jungles—so‐called urban life—was also characterized by microphobia and obsessive levels of personal hygiene, which reduced the diversity of human microbiomes even further, and made humans even more susceptible to disease. And, in colonizing ever more of the planetary surface, they destroyed important natural habitats providing key ecological services and exposed themselves to exotic animal pathogens that then evolved to infect humans. Some of these pathogens caused infections that rapidly spread across the globe, the so‐called pandemics. Vaccines were quickly developed that contained some of the infections (Brüssow, 2021, 2022), but immune system function progressively deteriorated as stresses on humans increased.

*Luca*: What sort of stresses?

*Granny*: One of these was of course the horror of realization that money lacks intrinsic value and that it cannot fix a dying biosphere: deterioration of the natural (real) world wreaked havoc on the health of the financial (imaginary) world and the value of money dropped precipitously. Eventually, a combination of global warming‐induced flooding, storms, forest fires and high ambient temperatures, starvation due to failing global food security, reducing availability of clean water, pollution, disease and societal breakdown due to fighting over ever‐diminishing resources, killed off the last humans, and with them, all other *phogs* and *fhogs*. One of the last remaining humans of the last *hog* generation, the *lhogg*, was heard to say: *last one to leave turn off the lights!*

*Luca*: But didn't anyone try to stop this madness? Just as we are diverse in our little crevice and have different opinions on how to solve our problems, surely, they must have had people with other views?

*Granny*: Yes, Pet, there were many humans who tried to save the planet (https://sdgs.un.org/2030agenda). Some campaigned energetically for sustainability and for a reduction in consumption. In particular, heroines, like Greta Thunberg, and heroes, like David Attenborough, launched impressive movements for reduction of practices that emit greenhouse gases and cause global warming. And especially microbiologists—a group of humans who studied us—were active in such campaigns because some microbes produce greenhouse gases and contribute to global warming, whereas others capture them and help mitigate against global warming (Cavicchioli *et al*., 2019). More generally, microbiologists tried to steer humans towards cooperation and synergies with microbes (Timmis and Ramos, 2021), instead of confrontation, through education (Timmis *et al*., 2019) and through persuasion of adults of the amazing potential of microbes to help solve surface biosphere problems (Timmis *et al*., 2017; https://sfamjournals.onlinelibrary.wiley.com/toc/17517915/2017/10/5). But all of this was ultimately in vain. And, despite the fact that it was once thought that humans might orchestrate only their own extinction (Dixon, 1981), global warming eventually made life impossible for all *phogs* and *fhogs*, and indeed, many of ourmicrobial relatives that lived on the surface of the planet.

Luca: Did *hogs* disappear without a trace?

Granny: Oh no! Humans, especially, also left an amazing legacy. Microbiologists taught us all about our inner workings, ecophysiology, HGT and how versatile and robust we are. And, although most of the amazing artistic and cultural works humans created have in the meantime turned to dust, some like Petra, the Taj Mahal, La Pieta, Abu Simbel, Machu Picchu, the Acropolis, the Colosseum, the Great Sphinx, the Great Wall of China, Venice, the Giant Buddha of Leshan, Angkor and the Alhambra, still stand as evidence of their creativity, artistry and ability to focus (https://en.wikipedia.org/wiki/Artificial_structures_visible_from_space). But even these are slowly disappearing because they too constitute food for microbes (Scheerer *et al*., 2009; Gaylarde and Baptista‐Neto, 2021), and eventually, we will consume them.

*Luca*: But Granny, if the planet surface became too hostile for *hogs*, how did we survive?

*Granny*: Well, darling. As you know, microbes are incredibly versatile and have evolved the most amazing abilities to thrive under the most adverse conditions, so we continue on the surface, albeit less joyfully than before. But, even when *hogs* were around and provided us with *hog* habitats and food, the majority of us still lived in the subsurface (Whitman *et al*., 1998). So, life goes on down here pretty much the way it always did: *slowly* (Hoehler and Jorgensen, 2013; Zinke *et al*., 2017). Because whereas time was perceived by humans as always being in short supply—the limiting factor for things humans regarded as being essential to do, often without any particular compelling reason other than their mortality—so they were always in a hurry, setting deadlines, imposing sanctions and punishments for being late and so forth, for us, time is a resource available in unlimited supply (Lloyd, 2021). And—something I have not told you so far (and this will be a teaser for another story)—we may have relatives on other planets. Because humans, in their rush to explore and colonize everything and every place, developed powerful rockets to send themselves and instrument payloads to explore other planets. And in so doing, they also transported us everywhere they went: they ferried us to new worlds. But of course, down here, we have no idea if our travelling cousins survived out there. And, à propos the possibility of our colonizing other planets, no one really knows whether or not our own origins lie on another planet, and whether we were ferried to Earth in a similar way, something humans called *panspermia* (Ginsburg *et al*., 2018).

*Luca*: Will we ever be able to evolve again as before and give rise to *hogs*?

*Granny*: This is a good question, darling, but very difficult to answer. We microbes are extremely diverse (Locey and Lennon, 2016), creative and resourceful and, when faced with a problem or opportunity, usually come up with a plethora of solutions, so I imagine we will. However, it is doubtful that we will adopt an evolutionary trajectory that will produce *bossfhogs* again. What we ultimately do will depend on what environmental conditions obtain in the future—what Nature allows—but I think that we will never again permit any surface life we create to develop brains and intelligence and the means to gain independence from our control and to uncouple from Nature. We, at least, are too smart to make the same mistake twice.

## Concluding remarks

Clearly, to avoid, or at least delay, a scenario like that depicted above, humanity must immediately institute radical changes that drastically reduce greenhouse gas emissions and other planet‐destroying activities, and single‐mindedly seek to develop and exploit new technologies to repair existing damage. Moreover, we must enter into more cooperative relationships with microbes and other life forms and, with them, devise new and transformative technologies to solve or mitigate current and future global, regional and local environmental problems. This will necessitate changes in mindsets (e.g., see Timmis and Ramos, 2021). The next 15 years of microbial biotechnology *must* largely be devoted to the discovery and development of new microbial technologies that contribute significantly towards sustainability. Hopefully, during this time, humankind, with microbial assistance, will reorient its developmental trajectory to one that is consistent with the survival of Earth's surface biosphere.

## Dedication

This piece is dedicated to the authors of the Sustainability Development Goals, campaigners for sustainability, especially Greta and David, and the microbiologists who showed how microbes are part of both the greenhouse gas emission problem and its solution. K. T. particularly expresses his gratitude to Ricardo Amils and Victor Parro for generously involving him in their exciting Iberian Pyritic Belt Subsurface Life adventure and revealing the fascinating life of Luca and Granny and their microbial relatives and friends, *very* slowly and peacefully playing out in crevices of solid rock in the deep subsurface.

## Conflict of interest

None declared.

## Funding Information

No funding information provided.
